# Smoking, Alcohol, and Betel Quid and Oral Cancer: A Prospective Cohort Study

**DOI:** 10.1155/2011/525976

**Published:** 2011-03-17

**Authors:** Wen-Jiun Lin, Rong-San Jiang, Shang-Heng Wu, Fun-Jou Chen, Shih-An Liu

**Affiliations:** ^1^Department of Otolaryngology, Taichung Veterans General Hospital, Chung-Kang Road, Taichung 40705, Taiwan; ^2^Graduate Institute of Integrated Medicine, China Medical University, Taichung 40402, Taiwan; ^3^Faculty of Medicine, School of Medicine, National Yang-Ming University, Taipei 11221, Taiwan

## Abstract

We aimed to investigate the association between smoking, alcoholic consumption, and betel quid chewing with oral cancer in a prospective manner. All male patients age ≥18 years who visited our clinic received an oral mucosa inspection. Basic data including personal habits were also obtained. A multivariate logistic regression model was utilized to determine relevant risk factors for developing oral cavity cancer. A total of 10,657 participants were enrolled in this study. Abnormal findings were found in 514 participants (4.8%). Three hundred forty-four participants received biopsy, and 230 patients were proven to have oral cancer. The results of multivariate logistic regression found that those who smoked, consumed alcohol, and chewed betel quid on a regular basis were most likely to develop cancer (odds ratio: 46.87, 95% confidence interval: 31.84–69.00). Therefore, habitual cigarette smokers, alcohol consumers, and betel quid chewers have a higher risk of contracting oral cancer and should receive oral screening regularly so potential oral cancer can be detected as early as possible.

## 1. Introduction

Smoking is one of the most important risk factors for developing oral cancers [1, 2]. Oral cancer is currently a major global health issue [3]. In developing countries, oral cavity cancer is estimated to be the third most common malignancy after cancer of the cervix and stomach [4]. Oral cancer has also been one of the top 10 causes of death from cancer since 1991 in Taiwan and the death toll for oral cancer in males has been rising at a surprising rate [5].

No significant advancement in the treatment of oral cancer has been found in recent years. Although better combinations of multidiscipline approach have improved the quality of life in oral cancer patients, the overall 5-year survival rate has not improved much over the past decades [6]. Therefore, primary prevention such as cessation of tobacco smoking and alcohols drinking along with early detection is necessary control procedures to improve the prognosis of oral cancer [7].

Other risk factors have been reported to be closely associated with oral cancers including alcoholic consumption, betel quid chewing [8], poor oral health [9], and human papilloma virus infection [10]. The incidence of oral cancer among patients who had the habit of tobacco smoking was 8.4 fold higher than that among patients who did not [8]. Another study also found the smokers had a 6.41-fold increase in the risk of contracting oral cancer [11]. However, few prospective cohort studies for the risk factors of developing oral cancer have been performed. Therefore, this study aimed to investigate the relationship between smoking, alcoholic consumption, and betel quid chewing and oral cancers in a prospective manner. The synergistic effect of smoking, alcoholic consumption, and betel quid chewing was also examined.

## 2. Materials and Methods

This study was conducted in Taichung Veterans General Hospital, a tertiary refer center in central portion of Taiwan. All male patients who visited our clinic age 18 or older were eligible for enrollment in current study. Those who were reluctant to join this study were excluded. Participants were first asked to describe their personal habits during the past 6 months, including tobacco use, alcohol consumption, and betel quid chewing. Those who smoked cigarettes, drank alcohol, or chewed betel quid only on special occasions such as wedding banquets, family reunions, or birthday parties were not considered as habitual users. Next, visual inspection of the oral cavity was performed under adequate lighting and with proper instruments. A nonhealing ulcer for more than 2 weeks, a persistent white or red lesion, a lesion that bleeds easily, or an irregular surface lesion inside the oral cavity were regarded as positive findings. Punch biopsy of abnormal lesions was performed after a detailed explanation. If the patient hesitated about further biopsy, followup was strongly recommended.

### 2.1. Statistical Analysis

This study used descriptive statistics for general data presentation. Comparisons of nominal or ordinal variables between the patients proven to have oral cancer and those without oral cancer were analyzed by the Chi-square test. Furthermore, relevant factors for contracting oral cancer were analyzed by a multivariate logistic regression model. All statistics were calculated by SPSS for Windows, version 10.1 (SPSS Inc., Chicago, IL, USA). Statistical significance was considered as *P* < .05.

## 3. Results

A total of 10,657 patients were enrolled in this study from March 2005 to December 2008. All were male, and their ages ranged from 18 to 96 years with an average age of 55.2 years (±18.6 years). Habitual smokers accounted for 21.3% (*N* = 2,268) of the studied population, whereas habitual drinkers and betel quid chewers accounted for 14.7% (*N* = 1,569) and 7.1% (*N* = 758), respectively. Among habitual smokers, 1,068 participants (47.1%) were smokers only, whereas 534 participants (23.5%) were also alcohol consumers, 146 participants (6.4%) had the habit of betel quid chewing additionally, and 520 participants (22.9%) were also alcohol consumers and betel quid chewers. The majority of betel nut chewers (87.9%, *N* = 666) were also smokers and only 6.5% (*N* = 49) were solely betel quid chewers. 

Five hundred fourteen participants (4.8%) were recorded to have positive lesions. Among those with positive lesions, 344 participants (66.9%) underwent oral cavity biopsy. Among those who received biopsy, 230 participants (66.9%) were proven to have oral cancer. One hundred seventy participants (23.9%) with abnormal oral lesions were lost to followup, and no further pathological report could be obtained. In order not to confound further analysis, we excluded those who had positive lesions yet no additional biopsy obtained during the follow-up period. Other descriptive statistics are presented in Table 1.

### 3.1. Bivariate Analysis

After dividing all the participants into two groups (those with and those without pathologically proven cancer), the oral cancer group consisted of 230 participants and the control group consisted of 10,257 participants. Comparisons of variables between the two groups are detailed in Table 1.

There were significant differences between the two groups based on the age (*χ* ^2^ value = 100.82, *P* < .001). Besides, there was also a significant difference between the two groups in personal habits such as smoking, alcohol consumption, and betel quid chewing.

### 3.2. Logistic Regression Model

Using the results of pathological examination as a dependent variable, a multivariate logistic regression model for exploring the relevant risk factors for developing oral cancer was created. We found that those aged 50 to 59 years were more likely to contract oral cancer when compared with those less than 40 years old (odds ratio (OR): 6.19, 95% confidence interval (CI): 3.62–10.58, *P* < .001). Furthermore, those who were habitual smokers, alcohol consumers, and betel quid chewers had the highest risk of developing oral cancer when compared with those who did not have these habits (OR: 46.87, 95% CI: 31.84–69.00, *P* < .001). The detailed results are shown in Table 2.

## 4. Discussion

The remarkable increase in the per capita consumption of tobacco and alcohol might be the reason why the incidence of oral cancer increased after 1915 in the United States and other regions around the world [12]. In Taiwan, the annual production of betel nut has also increased year by year since 1981 [13]. This explains why the incidence of oral cancer has rapidly increased in Taiwan and why prevention of oral cancer has become a major public health issue.

Smoking, alcoholic consumption, and betel quid chewing are well-known risk factors associated with oral cavity cancer. However, the estimation of relative risks for contracting oral cavity cancer mostly came from case-control studies [8, 11–13]. In current study, we conducted a prospective cohort study to avoid selective bias that inevitably exits in the case control study.

In this hospital-based study, 10,657 participants received oral cavity inspection and 514 participants (4.8%) were found to have abnormal mucosa lesions. The reported percentage of suspicious lesions in the literature ranges from 1.3% to 16.3% [14]. The differences between the results of this study and other studies may be explained by different studied populations. Among those in this study who were found to have abnormal lesions, 344 participants later received biopsy and 230 patients were proven to have malignancies. Therefore, the positive predictive rate of this study was 68.9%, which is comparable with that of other studies [15]. 

The prevalence of smoking in this study was 21.3%, which is comparable to that of previous studies. The prevalence of alcohol consumption and betel quid chewing in this study was also similar to that of previous studies conducted in Taiwan [13]. In addition, almost all betel quid chewers were smokers (666 out of 758 patients), which was a finding of previous studies conducted in Taiwan [8, 13]. Therefore, the composition of the population in this hospital-based study is considered to be similar to the general male population in Taiwan.

Tobacco contains N-nitroso compounds, well-known carcinogens, which play a key role in the malignant transformation of oral cancer [16]. Other tobacco carcinogens include the polycyclic aromatic hydrocarbons (PAH) and 4-(methylnitrosoamino)-1-(3-pyridyl)-1 butanone (NNK). They can induce specific mutations, particularly G:T transversions [17]. Chronic exposure to tobacco carcinogens in the oral mucosa causes genetic changes in the epithelial cells. Cumulative genetic changes lead to genomic instability, development of premalignant lesions, and eventually invasive carcinoma. Tobacco may also induce proliferative activity through activation of the EGFR receptor and its downsteam mechanisms. This activates cyclin D1, leading to greater proliferative activity and higher frequency of mutations, thus rendering the cell more susceptible to permanent genetic changes, that in turn may give rise to genomic instability and invasive carcinoma [18]. 

According to the annual report by the Taiwan Cancer Registry System, the median age of diagnosis for oral cancer is 51.0 years [5]. Consequently, it is easy to understand why those aged 50–59 years in this study were most likely to develop oral cancer. On the other hand, only 17 out of 2,368 patients (0.7%) under the age of 40 in this study were proven to have oral cancer. Thus, it might be reasonable to start oral mucosa screening of males when they reach the age of 40.

Ko et al. in a case-control study showed that the incidence of oral cancer was 123-fold higher in those who smoked, drank alcohol, and chewed betel quid than in abstainers [8]. However, selection bias inevitably exists in case-control studies. In this study, it was interesting to note that those who drank only alcohol did not have an increased risk of developing oral cancer. A possible explanation might be that we did not collect quantitative data on alcohol consumption. In addition, different types of alcoholic beverages have different effects on the development of oral cancers [11]. Previous studies found evidence of the synergistic effects of smoking, drinking, and betel quid chewing on the risk of developing oral cavity cancer [8, 11]. This might be explained by the fact that betel quid chewers are proportionately heavier smokers, which was also true in the current study. Another study proposed that the alcohol might facilitate the passage of carcinogens through cellular membranes. In addition, alcoholic consumption enhanced liver metabolising activity in both humans and experimental animals and might, therefore, activate carcinogenic substances. Furthermore, alcohol might alter intracellular metabolism of the epithelial cells at the target site [19]. As a result, the oral mucosa was more vulnerable to carcinogens brought by smoking and betel quid chewing.

There were certainly some limitations in this study. First, the external validity of the findings is limited because the study was conducted at a single institution and only included patients visiting our clinic for otolaryngological problems. Second, we did not obtain information regarding quantities of consumption. Consequently, the dose-response relationship of these three risk factors for oral cancer cannot be demonstrated. Lastly, we only recruited male patients. In future studies, it would be useful to compare these data with those obtained from female patients.

## 5. Conclusion

In this prospective cohort study, we found a strong relationship between smoking, alcoholic consumption, and betel quid chewing in oral cancer. Synergistic effects endured patients with all the above habits had an over 40-fold higher risk of developing oral cavity cancer than patients who abstained. Therefore, we recommend those aged ≥40 years who are habitual cigarette smokers, alcohol consumers, and betel quid chewers undergo oral mucosa screening regularly so that potential oral cancer can be identified as early as possible.

## Figures and Tables

**Table 1:** Descriptive and bivariate analyses of the studied population.

Variables	No. of patients (% in column) (*N* = 10,657)	Oral cavity cancer*		*P* value
		Yes	No	
		No. of patients (%)	No. of patients (%)	
Age				
18–39 years	2368 (22.2%)	17 (0.7%)	2314 (99.3%)	<.001
40–49 years	1879 (17.6%)	61 (3.3%)	1777 (96.7%)	
50–59 years	2118 (19.9%)	94 (4.5%)	1977 (95.5%)	
≥60 years	4292 (40.3%)	58 (1.4%)	4189 (98.6%)	
Habitual smoker				
Yes	2268 (21.3%)	174 (8.0%)	1993 (92.0%)	<.001
No	8389 (78.7%)	56 (0.7%)	8264 (99.3%)	
Habitual drinker				
Yes	1569 (14.7%)	138 (9.2%)	1356 (90.8%)	<.001
No	9088 (85.3%)	92 (1.0%)	8901 (99.0%)	
Habitual betel quid chewer				
Yes	758 (7.1%)	126 (18.3%)	564 (81.7%)	<.001
No	9899 (92.9%)	104 (1.1%)	9693 (98.9%)	
Abnormal mucosa lesion				
Yes	514 (4.8%)	230 (66.9%)	114 (33.1%)	<.001
No	7974 (95.2%)	0 (0%)	10143 (100%)	

*Those with abnormal mucosa lesions but no further biopsy were excluded (*N* = 10,487).

**Table 2:** Multivariate logistic regression model of risk factors for developing oral cancer.

Variables	No. of patients (*N* = 10,487)	Odds ratio	95% Confidence Interval		*P* value
			Lower limit	Upper limit	
Age					
18–39 years^†^	2330	1.00			<.001
40–49 years	1838	3.68	2.11	6.41	<.001
50–59 years	2071	6.19	3.62	10.58	<.001
≥60 years	4247	3.66	2.09	6.43	<.001
Personal habits					
None^†^	7775	1.00			<.001
Smoking only	1040	5.13	3.17	8.32	<.001
Alcohol consumption only	464	1.33	0.48	3.74	.584
Betel quid chewing only	43	11.95	3.54	40.33	<.001
Smoking + alcohol	518	9.88	6.05	16.12	<.001
Smoking + betel quid	135	26.56	14.52	48.58	<.001
Alcohol + betel quid	38	21.84	8.04	59.36	<.001
Smoking + alcohol + betel quid chewing	474	46.87	31.84	69.00	<.001

^†^Reference group.
